# Supplementary material for: Evaluating the predictive power of pump-probe imaging contrast of melanin for metastatic outcome: A 71-patient study
Source: ArXiv. 2026 Sep 8:arXiv:2609.08814v1. Preprint. [Version 1] (PMC13622873)
Supplement: Supplementary file 1 [file NIHPP2609.08814v1-supplement-1.pdf]

Supplementary Information

Evaluating the predictive power of pump-probe imaging contrast of melanin for metastatic outcome

S1 Laser Characterization

The temporal and spatial characteristics of the pump and probe beams were measured before imaging sessions to ensure consistent data quality across the full acquisition period, which spanned several years.

S1.1 Pulse widths

Pulse widths were determined from a TA curve of a Rhodamine 6G solution, whose transient absorption at these wavelengths is dominated by two-photon absorption. Because this process is instantaneous relative to the pulse duration, the measured TA curve approximates the intensity cross-correlation of the pump and probe pulses, from which the individual pulse widths are estimated assuming Gaussian pulse envelopes.

For $\lambda_{\text{pump}} = 770\,\text{nm}$ and $\lambda_{\text{probe}} = 730\,\text{nm}$ the average pulse width was $\bar{t}_{\text{pulse}} = (77 \pm 3)\,\text{fs}$, with a minimum of 71 fs and a maximum of 85 fs. For $\lambda_{\text{pump}} = 730\,\text{nm}$ and $\lambda_{\text{probe}} = 810\,\text{nm}$ the average pulse width was $\bar{t}_{\text{pulse}} = (66 \pm 4)\,\text{fs}$, with a minimum of 58 fs and a maximum of 69 fs. Per-session measurements are shown in Figure S1.

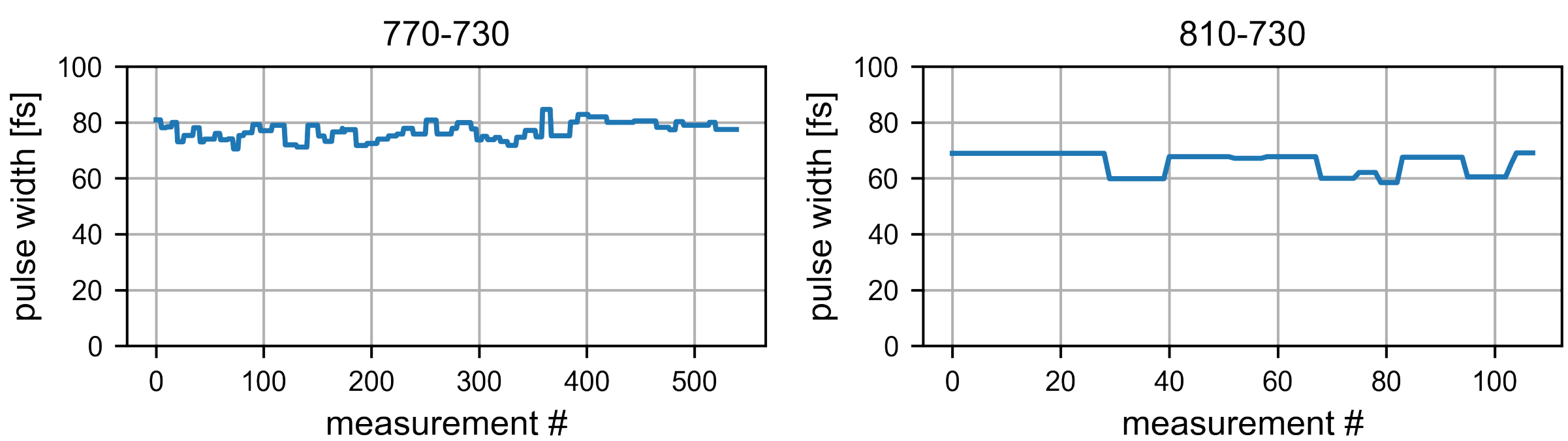

Figure S1: Cross-correlation measurements across the acquisition period. Pulse widths were extracted from the two-photon absorption response of Rhodamine 6G, assuming Gaussian pulse envelopes, for the 770/730 and 730/810 wavelength combinations.

S1.2 Spatial overlap.

To verify spatial overlap, the three-dimensional focus positions of the pump and probe beams were independently localized by imaging a 500 nm-diameter polystyrene bead via two-photon fluorescence. Beam alignment was iteratively adjusted until the two focal positions agreed to within half a standard deviation of the localization uncertainty. Note that in an ideal point-spread function measurement a sub-diffraction limit sized bead is imaged. Here, however, we only want to co-localize the two beams which can be achieved with larger beads, too.

S1.3 Polarization control.

For 770/730 acquisitions, the half-wave plate ($\lambda/2$) was aligned so that both beams were parallel polarized, and a polarizer (not shown in the setup sketch of the main text) was inserted before the microscope objective to further ensure parallel polarization. For 730/810 acquisitions, each ROI was imaged twice: the half-wave plate was first set for parallel polarization and then rotated so that pump and probe were perpendicular. No polarizer was used in this configuration.

S2 Surgical Ink Removal

Surgical ink used to mark tumor boundaries during excision occasionally persists in the unstained sections and produces a strong pump-probe signal unrelated to melanin, which must be removed prior to analysis. An example is shown in Figure S2. In the unstained section (a), the blue-tinted regions indicated by black arrows correspond to residual ink, while the brown areas are melanin. In the intensity projection of the corresponding PPM stack (b), the ink signal substantially exceeds that of melanin in magnitude. The blue and orange boxes in (b) mark ink- and melanin-dominated regions respectively; their average TA curves (c) show clearly distinct dynamics. All stacks were visually screened and regions containing surgical ink were manually cropped.

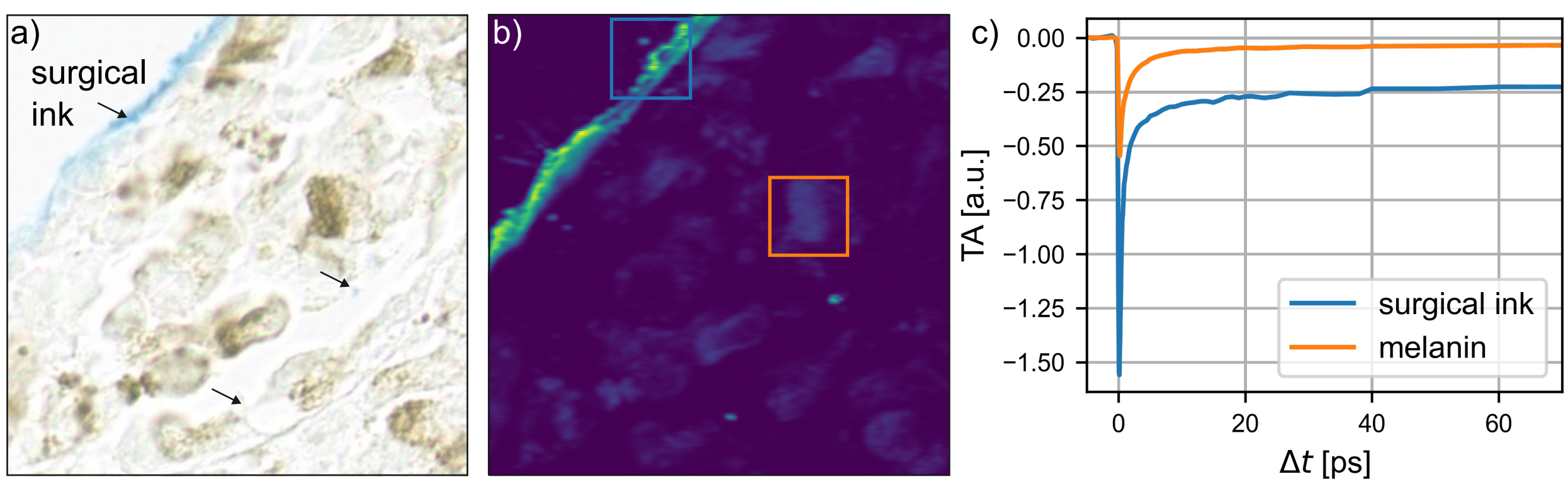

Figure S2: a) Unstained tumor section with residual blue surgical ink (black arrows) and melanin. b) Intensity projection of the PPM image stack of the adjacent unstained section; the ink produces a much stronger signal than melanin. c) Average TA curves from the ink-dominated (blue) and melanin-dominated (orange) regions boxed in b).

S3 Baseline Subtraction and Intensity-Based Masking

Excited-state dynamics with lifetimes much longer than the 12.5 ns inter-pulse spacing of the 80 MHz laser manifest as a constant offset in the TA signal. This offset was removed by averaging the images acquired at negative time delays ($\Delta t \leq -1$ ps) and subtracting the result from the entire stack. This corresponds to the first five images for 770/730 acquisitions and the first four for 730/810 acquisitions.

To exclude regions with little or no melanin signal, an intensity mask was constructed for each stack by summing the absolute signal across all time delays, applying a Gaussian smoothing filter with a kernel size of 2 pixels, and thresholding at a value of 2 (in arbitrary units). This threshold was chosen empirically to balance rejection of background regions against retention of genuine melanin signal.

S4 Excited-State Model: Functional Forms

The main text describes the excited-state model conceptually. The explicit functional forms are provided here. Each contribution to the TA signal is convolved with the instrument response function of the microscope, which is itself the convolution of the pump and probe temporal envelopes. Assuming Gaussian envelopes, this double convolution is equivalent to a single convolution with a Gaussian of width $t_p = \sqrt{t_{\text{pump}}^2 + t_{\text{probe}}^2}$.

For a delayed process with lifetime τ, the resulting TA signal as a function of time delay Δt is

$$s_{\text{delayed}}(\Delta t, \tau) = \tfrac{1}{2}\, e^{-\Delta t/\tau + t_p^2/(2\tau^2)} \left[1 + \text{erf}\left(\frac{\Delta t}{\sqrt{2}\, t_p} - \frac{t_p}{\sqrt{2}\, \tau}\right)\right]. \tag{S1}$$

When the lifetime is much longer than the acquisition window, the signal is approximated by the limit $\tau \to \infty$,

$$s_\infty(\Delta t) = \tfrac{1}{2}\left[1 + \text{erf}\left(\frac{\Delta t}{\sqrt{2}\, t_p}\right)\right], \tag{S2}$$

and for an instantaneous process the signal is Gaussian,

$$s_{\text{inst}}(\Delta t) \propto \frac{1}{\sqrt{2\pi\, t_p^2}}\, e^{-\Delta t^2/(2t_p^2)}. \tag{S3}$$

S5 Polarization Decomposition

Figure S3 illustrates the polarization-based decomposition applied to the 730/810 acquisitions. The bipolar TA curves measured with parallel and perpendicular pump-probe polarization are linearly combined, using the relations given in the main text, into the two unipolar GSB and ESA components.

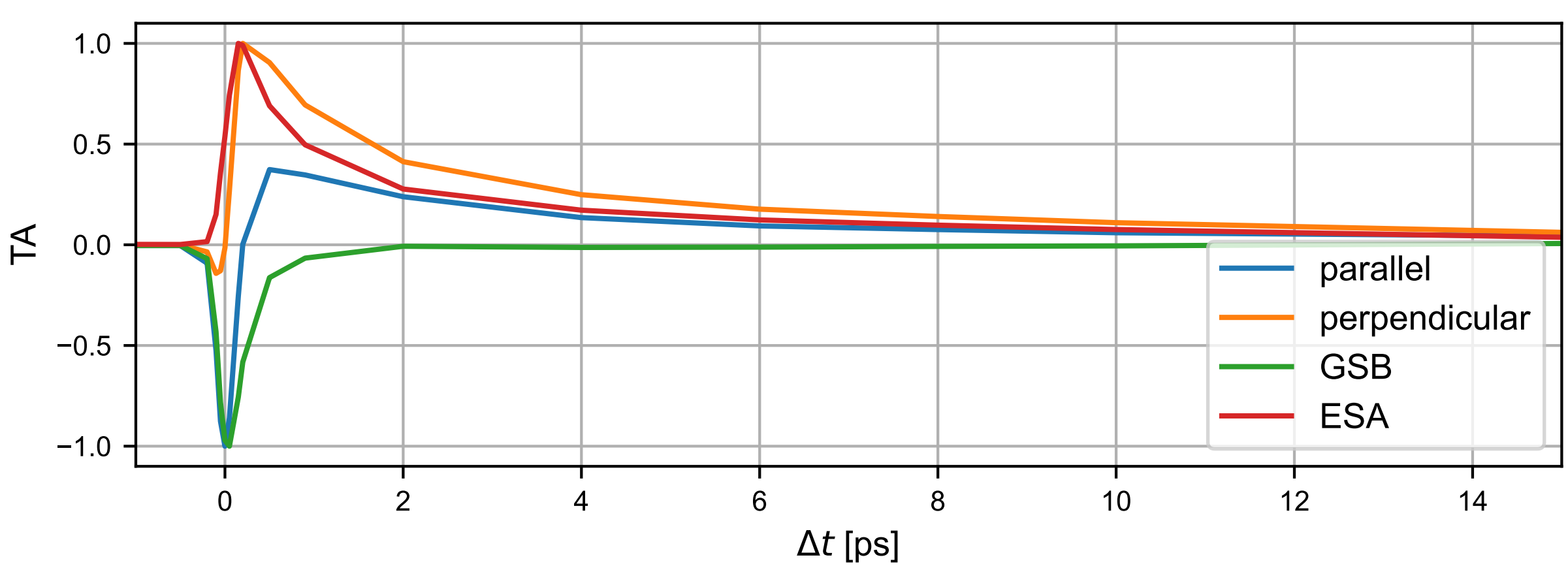

Figure S3: Polarization-based decomposition of pump-probe signals into GSB and ESA components. The bipolar TA curves measured with parallel (blue) and perpendicular (orange) pump-probe polarization are linearly combined to yield the unipolar GSB (green) and ESA (red) dynamics.

S6 Classifier Performance Across Bin Sizes

The main text reports classifier performance at the best-performing bin size for each wavelength combination. Figures S4, S5 and S6 show the full sweep over $l_{px} \in \{16, 32, 64, 128, 256, 512\}$ pixels.

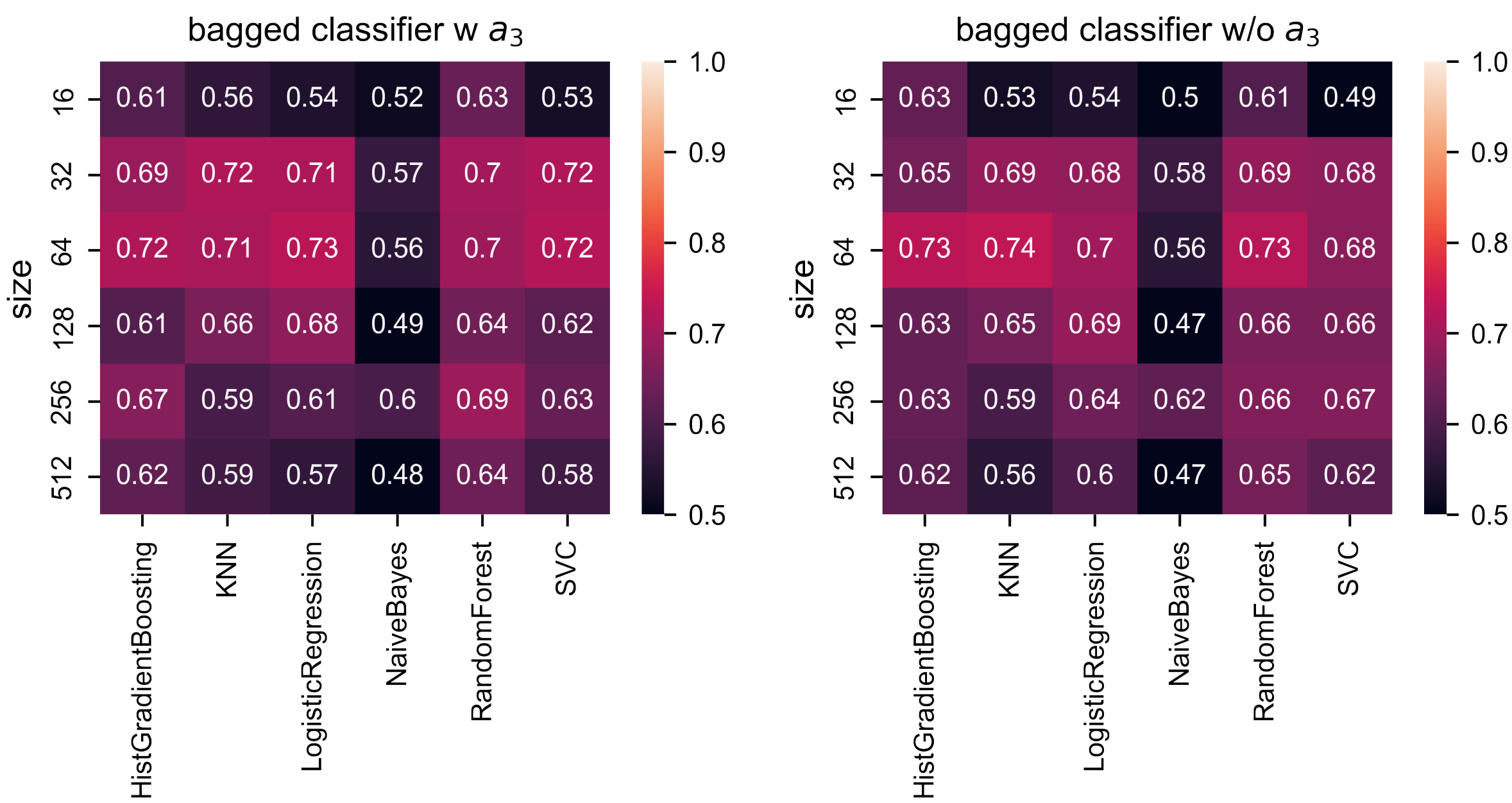

Figure S4: Patient-level test accuracy of the six classifiers on the 770/730 dataset for all bin sizes l_{px} under bagging strategies including a_3 (labeled "w a_3") and excluding a_3 (labeled "w/o a_3")

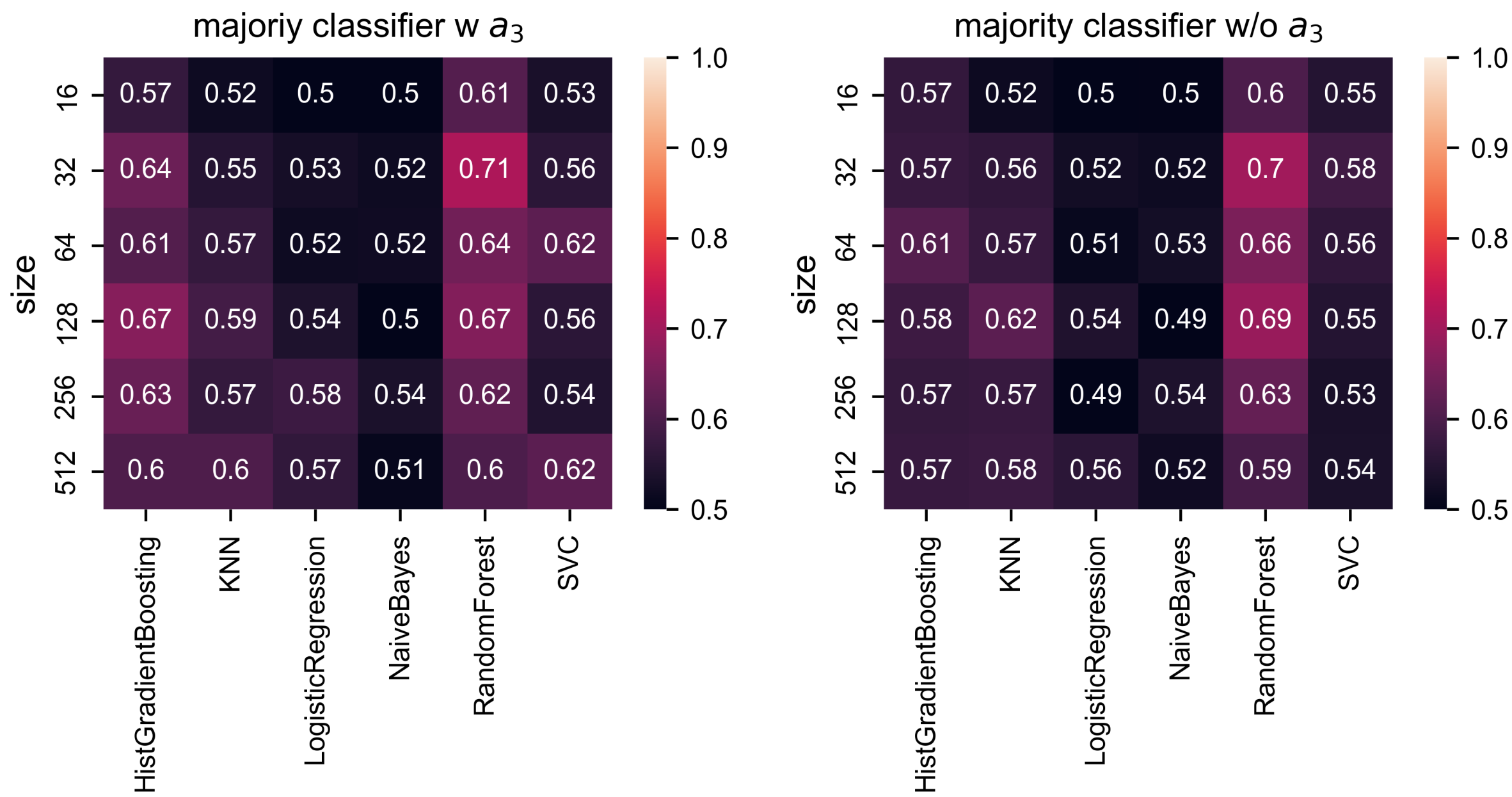

Figure S5: Patient-level test accuracy of the six classifiers on the 770/730 dataset for all bin sizes l_{px} under majority voting strategies including a_3 (labeled "w a_3") and excluding a_3 (labeled "w/o a_3")

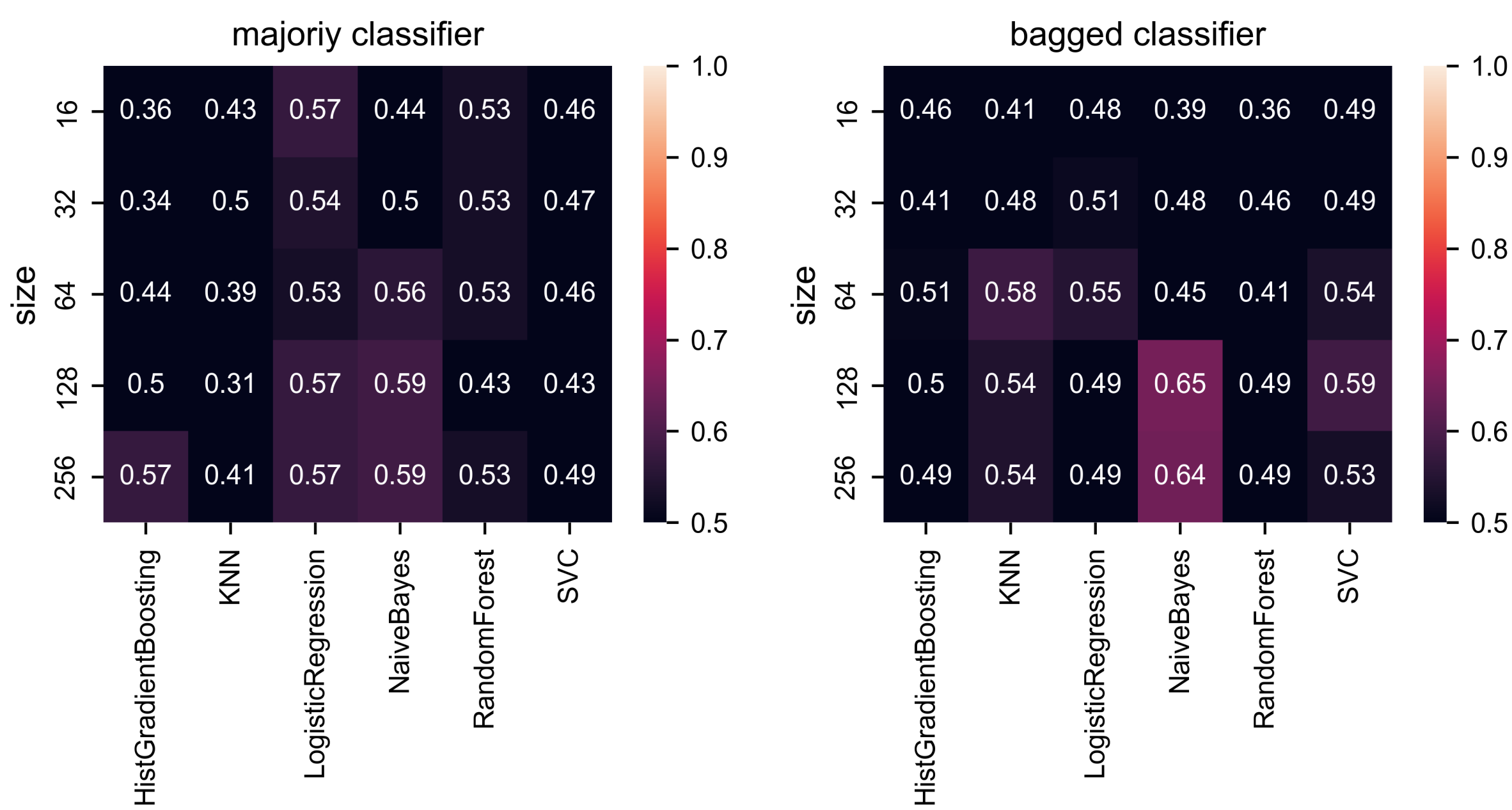

Figure S6: Patient-level test accuracy of the six classifiers on the 730/810 dataset for all bin sizes l_{px} under majority voting strategy (left) and bagging strategy (right)
